# Virus induced gene silencing confirms oligogenic inheritance of brown stem rot resistance in soybean

**DOI:** 10.3389/fpls.2023.1292605

**Published:** 2024-01-08

**Authors:** Chantal E. McCabe, Lori M. Lincoln, Jamie A. O’Rourke, Michelle A. Graham

**Affiliations:** ^1^ United States Department of Agriculture, Agricultural Research Service (USDA-ARS), Corn Insects and Crop Genetics Research Unit, Ames, IA, United States; ^2^ Department of Agronomy, Iowa State University, Ames, IA, United States

**Keywords:** brown stem rot, *Phialophora gregata*, soybean, receptor-like protein, virus induced gene silencing, Rbs

## Abstract

Brown Stem Rot (BSR), caused by the soil borne fungal pathogen *Phialophora gregata*, can reduce soybean yields by as much as 38%. Previous allelism studies identified three Resistant to brown stem Rot genes (*Rbs1, Rbs2*, and *Rbs3*), all mapping to large, overlapping regions on soybean chromosome 16. However, recent fine-mapping and genome wide association studies (GWAS) suggest *Rbs1, Rbs2*, and *Rbs3* are alleles of a single *Rbs* locus. To address this conflict, we characterized the *Rbs* locus using the Williams82 reference genome (Wm82.a4.v1). We identified 120 Receptor-Like Proteins (RLPs), with hallmarks of disease resistance receptor-like proteins (RLPs), which formed five distinct clusters. We developed virus induced gene silencing (VIGS) constructs to target each of the clusters, hypothesizing that silencing the correct RLP cluster would result in a loss of resistance phenotype. The VIGS constructs were tested against *P. gregata* resistant genotypes L78-4094 (*Rbs1*), PI 437833 (*Rbs2*), or PI 437970 (*Rbs3)*, infected with *P. gregata* or mock infected. No loss of resistance phenotype was observed. We then developed VIGS constructs targeting two RLP clusters with a single construct. Construct B1a/B2 silenced *P. gregata* resistance in L78-4094, confirming at least two genes confer *Rbs1*-mediated resistance to *P. gregata*. Failure of B1a/B2 to silence resistance in PI 437833 and PI 437970 suggests additional genes confer BSR resistance in these lines. To identify differentially expressed genes (DEGs) responding to silencing, we conducted RNA-seq of leaf, stem and root samples from B1a/B2 and empty vector control plants infected with *P. gregata* or mock infected. B1a/B2 silencing induced DEGs associated with cell wall biogenesis, lipid oxidation, the unfolded protein response and iron homeostasis and repressed numerous DEGs involved in defense and defense signaling. These findings will improve integration of *Rbs* resistance into elite germplasm and provide novel insights into fungal disease resistance.

## 1 Introduction

Pests and pathogens have a significant impact on plant yields, affecting the economic well-being of entire nations and individual livelihoods (Wrather et al., 2001). Globally, pathogens are responsible for up to 32% of soybean yield loss (Savary et al., 2019). The fungal pathogen *Phialophora gregata*, which causes brown stem rot (BSR), led to a reduction of 3.176 million bushels in soybean yield in the U.S.A in 2022 (Allen et al., 2023). While BSR is agronomically important, the available methods for managing it are limited to genetic resistance and long-term crop rotation (Adee et al., 1997). Despite genetic resistance being the most cost-effective means of preventing BSR-related yield losses, the characterization of novel traits for BSR disease resistance remains challenging. Disease symptoms in the stem take several weeks to develop and, if present, foliar symptoms can be confused with other diseases or nutrient stress.

Three dominant, independent BSR resistance genes have been identified using allelism studies: *Rbs1, Rbs2*, and *Rbs3* (Sebastian and Nickell, 1985; Hanson et al., 1988; Willmont and Nickell, 1989). All three *Rbs* genes were mapped to large, overlapping regions on soybean chromosome 16 (Lewers et al., 1999; Klos et al., 2000; Bachman et al., 2001). Given the linkage of the three genes on chromosome 16, Bachman and Nickell (Bachman and Nickell, 2000) proposed that the three *Rbs* loci interact with a fourth locus to confer resistance. In contrast, recent fine mapping and genome wide association studies (GWAS) mapped *Rbs1, Rbs2*, and *Rbs3* to the same 41 kb interval within the historical *Rbs* loci, leading the authors to propose a single BSR resistance gene (Rincker et al., 2016a; Rincker et al., 2016b).

Given the confusion surrounding BSR resistance, our previous work (McCabe et al., 2018) examined the 687 high confidence genes in version 2 of the Williams82 reference genome (Schmutz et al., 2010) located between simple sequence repeat (SSR) markers Satt215 and Satt431 on chromosome Gm16. This region spans all mapped *Rbs* loci on Gm16. Annotation of these genes identified 107 Receptor-Like Proteins (RLPs), 18 nucleotide-binding site (NBS) leucine-rich repeat (LRR) resistance gene (R-gene) homologs and two receptor-like kinases (RLKs). While RLPs have diverse biological functions, careful alignment of the *Rbs* RLPs identified conserved cysteine residues specific to RLPs associated with plant defense (Fritz-Laylin et al., 2005). The alignment divided the *Rbs* RLPs into five distinct classes, B1 to B5 (McCabe et al., 2018), which could be associated with different *Rbs* QTL. The *Rbs* RLPs had homology to other RLP resistance genes, such as the apple *HcrVf* genes (apple scab), the tomato *Cf* genes (*Cladosporium fulvum*), *Ve* genes (Verticillium wilt) and *LeEIX* genes (*Trichoderma viride*), and the Arabidopsis *RPP27* gene (*Peronospora parasitica*), all thought to encode Pattern Recognition Receptors (PRRs) that recognize extracellular damage caused by pathogen attack or pathogen effectors directly and mediate immune responses (Ngou et al., 2022). In addition, the RLPs contain many hallmarks of resistance genes, including conserved resistance motifs, physical clustering of genes, and evidence of unequal recombination. The high proportion of RLPs in this region highlights the complexity of the *Rbs* loci and may explain difficulties in mapping resistance. Layering the RLPs with historical mapping and expression data suggested that different clusters of RLPs were associated with the *Rbs1*, *Rbs2*, and *Rbs3* loci.

To understand the genes and gene networks underlying BSR resistance, a new approach was needed. Therefore, we took advantage of virus induced gene silencing (VIGS, Zhang et al., 2009) which has successfully been used in soybean to characterize candidate disease resistance genes for soybean rust (Meyer et al., 2009; Pedley et al., 2019) and soybean cyst nematode (Liu et al., 2012), demonstrating effective silencing of resistance genes for diverse pathogens and in distant plant tissues. VIGS can be targeted to a unique portion of a single gene or to a conserved region shared by multiple genes (Zhang et al., 2009). We developed VIGS constructs representing each of the RLP clusters we previously identified (McCabe et al., 2018). We hypothesized that silencing of the correct RLP(s) in the appropriate genetic background would result in loss of resistance to *P. gregata.* RNA-seq analyses of plants with a loss of resistance due to VIGS and control plants could then be used to identify the gene networks responsible for resistance. If successful, this approach would provide much needed information on the genetics of BSR resistance and new opportunities for soybean improvement.

## 2 Materials and methods

### 2.1 Updating the *Rbs* loci and candidate RLPs

**Figure 1:**
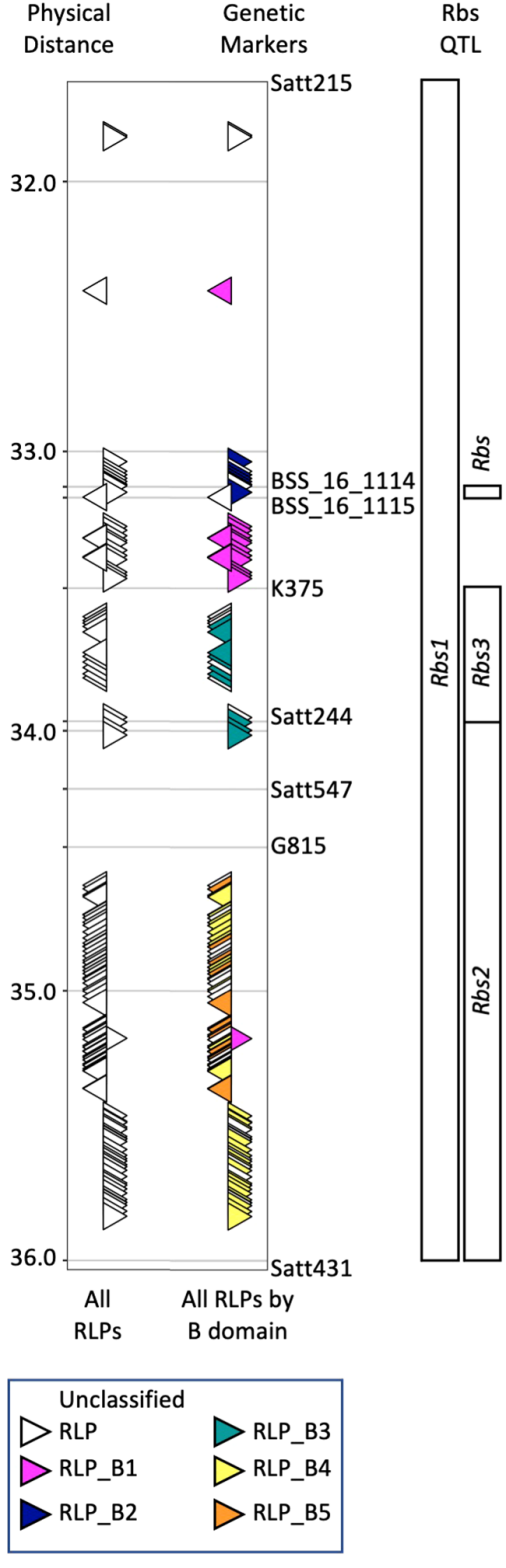
Organization of RLPs within previously identified *Rbs* loci on chromosome 16. Map locations of *Rbs1* and *Rbs2* were determined by Bachman et al. (2001). Map location of *Rbs3* was determined by Lewers et al. (1999) and Klos et al. (2000). Rincker et al. (2016a; 2016b) used fine mapping and GWAS to propose a single *Rbs* locus. Genetic markers used in these studies were used to identify the corresponding region from the reference genome (Wm82.a4.v1). QTL and genetic markers are shown to the right of the figure panel, while physical distance is shown to the left. The position and orientation of 120 predicted RLPs (white triangles) relative to all *Rbs* QTL is shown on the left side (All RLPs). Alignment of RLPs with full-length B-domains was used to color code RLPs by class based on similar B-domains (B1-B5). RLPs with partial or lacking B-domains could not be assigned to a class and are designate as unknown (UK). VIGS constructs were developed to target clusters of RLPs identified by their B domain.

McCabe et al. (2018) used historical mapping data and the Willams82 reference genome sequence [version Wm82.a2.v1, (Schmutz et al., 2010)] to demonstrate *Rbs1*, *Rbs2* and *Rbs3* were located within overlapping regions of soybean chromosome 16. To update this information, BLASTN (Camacho et al., 2009) was used to compare the marker sequences (Satt215, BSS_16_114, BSS_16_115, K375, Satt244, G815 and Satt431) used to map the different *Rbs* loci to version 4 of the Williams82 reference genome (Wm82.a4.v1). All three *Rbs* loci were located with a 4.03 Mb region on chromosome 16 (Gm16: 29,140,388 to 33,171,315, **Figure 1**). BLASTP (Camacho et al., 2009) was used to compare the protein sequences of the 679 genes located within the updated *Rbs* loci (Wm82.a4.v1) to the 107 RLPs previously identified (Wm82.a2.v1, McCabe et al., 2018). Similarly, the SoyBase Gene Annotation Tool^1^ was used to confirm 120 predicted RLPs (**Supplementary Table 1**). The 120 RLPs were aligned using Jalview version 2.11.2.7 (Waterhouse et al., 2009) and T-Coffee (Notredame et al., 2000). Comparison between the Wm82.a2.v1 clusters and the current alignment was used to maintain cluster identification between genome versions. While Williams82 is susceptible to *P. gregata*, the Williams82 genome sequence coupled with VIGS has been used successfully to identify candidate resistance genes for soybean rust (Meyer et al., 2009; Pedley et al., 2019) and soybean cyst Nematode (Liu et al., 2012).

### 2.2 Generating and screening VIGS constructs

Target gene fragments from each RLP class (157 to 516 basepairs) were amplified from the genomic DNA of resistant genotype of interest (**Supplementary Table 2**). Constructs B1a, B1b, B2, B3, B4 and B5 contained a fragment from a single gene of interest. Constructs B1a/B2, B1b/B2, B4/B5 each contained fragments from two genes of interest. Fragments used for B1a/B2, B1b/B2 and B4/B5 were the same as used B1a, B1b, B4 and B5. Fragments were cloned into RNA2 of the Bean Pod Mottle Virus (BPMV) VIGS vector in the antisense orientation following the protocol described in by Whitham et al. (2016). To target two distinct genes in the same VIGS construct, the Gibson Assembly Method (Gibson et al., 2009) was used to ligate PCR products prior to insertion into the BPMV VIGS vector (**Supplementary Table 2**). The orientation and identity of all VIGS inserts were confirmed by sequencing using a multiple cloning site vector-specific primer (Whitham et al., 2016). Since BPMV has a bipartite genome consisting of two RNAs (Zhang et al., 2009), BPMV RNA1 and the recombinant RNA2 DNA clones were used for particle bombardment on leaves of 14 day old Williams82 seedlings (Whitham et al., 2016). BPMV infection was confirmed with a BPMV ELISA antibody test (Agdia, Elkhart, IN) at three weeks, and BPMV-infected leaf tissue was collected and lyophilized at five weeks. To generate enough inoculum for the VIGS experiments, the bombarded, lyophilized leaf tissue corresponding to each VIGS construct was used for rub inoculation. The unifoliates of ten-day old Williams82 seedlings were dusted with carborundum and rub inoculated with the corresponding VIGS tissue suspended in 50 mM potassium phosphate buffer, pH 7.0. Three weeks after infection, BPMV infection was confirmed with a BPMV ELISA antibody test (Agdia, Elkhart, IN), and positive leaf tissue was collected and lyophilized at five weeks. All plants were grown in Metro-Mix 900 potting soil (Sun Grow Horticulture, Agawam, MA) in a growth chamber maintained at a constant 19 ± 1.5°C with a 16-h photoperiod. Plants were watered daily and fertilized weekly with a 24-8-16 fertilizer mixture.

To determine the effect of each VIGS construct on resistance, BSR resistant genotypes L78-4094 (*Rbs1*), PI 437833 (*Rbs2*), or PI 437970 (*Rbs3)* were rub inoculated with lyophilized leaf tissue corresponding to each VIGS construct. Controls included control plants inoculated with lyophilized leaf tissue from BPMV empty vector (EV) plants and control plants inoculated with the 50 mM potassium phosphate buffer used to deliver lyophilized leaf tissue (no BPMV). Four to six seedlings of each genotype were infected with each BPMV VIGS construct. 48 hours after BPMV infection, plants were stab inoculated at the soil line with *P. gregata*, isolate *Oh_2-3_* (Lewers et al., 1999) at a concentration of 2.7 × 10^7^ spores/ml in a water agar slurry, as described by McCabe et al. (2018). Soybean genotype Corsoy79 was included as a susceptible check for *P. gregata* infection. An additional replicate included plants mock infected with water agar. Three weeks after rub inoculation, BPMV infection was confirmed with a BPMV ELISA antibody test (Agdia, Elkhart, IN). Plants were evaluated for BSR resistance or susceptibility five weeks after infection. Subsequent screenings were conducted on a minimum of three independent replicates.

### 2.3 RNA-seq analyses of the VIGS plants with a loss of resistance phenotype

Following a loss of *P. gregata* resistance for plants treated with the B1a/B2 VIGS construct, an additional set of L78-4094 (*Rbs1*) seedlings was grown using the growth conditions described above. Ten-day old seedlings were rub inoculated with lyophilized tissue of either the B1a/B2 VIGS construct or EV. Forty-eight hours after rub inoculation, plants were either infected with *P. gregata* or a mock treatment consisting of only water agar, as described above. One week after infection with *P. gregata* or mock treatment, leaf, stem, and root tissues were collected from four plants in each of the four treatments: empty vector-*P. gregata*, empty vector-mock treatment, B1a/B2-*P. gregata*, B1a/B2-mock treatment. The first fully expanded trifoliate leaf, the stem section between the cotyledon and the unifoliate, and the whole root were collected. Samples were flash frozen in liquid nitrogen and stored at −80°C. Three weeks after infection, an ELISA was conducted on the remaining plants of each of the four treatments. These plants were phenotyped at five weeks after infection to verify expected BSR symptoms using foliar and stem severity measurements, as described by McCabe et al. (2018).

Flash frozen tissue was ground with a Qiagen® TissueLyser® (Qiagen®, Germantown, MD) and RNA was extracted using a Qiagen® RNeasy® Plant Mini Kit (Qiagen®, Germantown, MD) following the manufacturer’s recommended protocol. Contaminating DNA was removed with an Ambion® TURBO DNA-free kit™ (Ambion®, Austin, TX) and RNA was purified and concentrated using a Qiagen® RNeasy® MinElute Cleanup Kit (Qiagen®, Germantown, MD). The 48 samples, one genotype x two VIGS constructs (B1a/B2 and EV) x three plant tissues (leaves, stems and roots) x two treatments (infection with *P. gregata* or mock infection) x 4 plant replicates, were assessed for purity and quantification using a Thermo Fisher Scientific® NanoDrop ND-1000 Spectrophotometer (Thermo Fisher Scientific®, Waltham, MA, USA). Library preparation and 150 bp single end sequencing was conducted with 1 µg of total RNA using the Illumina NovaSeq 6000 platform (Illumina®, San Diego, CA) at the Iowa State University DNA Facility.

### 2.4 Bioinformatic and statistical analyses

Raw reads from 48 samples were inspected with FASTQC^2^ to confirm sequence quality and quantity. In addition, raw reads were analyzed with FASTQ Screen (Wingett and Andrews, 2018) to assess read numbers relative to version 4 of the Williams82 genome sequence [Wm82.a4.v1, (Schmutz et al., 2010)] and BPMV RNA1 and RNA2 (GenBank Accessions GQ996952 and GQ006949). Sequences from 22 RNA samples that had fewer than 4% of reads mapping to the BPMV vector sequences were removed from further analyses. Removed samples included: EV mock-infected (two samples each from leaves, stems and roots), EV *P. gregata* infected (two samples each from leaves, stems and roots), B1a/B2 mock infected (one leaf sample, two stem samples, one root sample), and B1a/B2 *P. gregata* infected (two samples each from leaves, stems, and roots). For the remaining 26 samples, the 150 base pair reads from the remaining samples were trimmed to remove adaptor sequences (Scythe^3^), sequencing artifacts (FASTX trimmer^4^), and low-quality bases (Sickle^5^). TopHat version 2.0.3 (Trapnell et al., 2009) was used to align reads to version 4 of the Williams82 reference genome sequence [Wm82.a4.v1, (Schmutz et al., 2010)]. Samtools (Li et al., 2009) was used to remove unreliably mapped reads with a mapping score < 1. For each tissue (leaves, stems or roots), mapping files (bamfiles) were imported into the statistical program R^6^ using Rsamtools^7^. The gene feature file (GFF) corresponding to the reference genome was imported using rtracklayer (Lawrence et al., 2009). The number of reads per sample aligning to each gene were counted using summarizeOverlaps^8^, and a count table for all predicted genes within a tissue was generated.

Given the sequence similarity of the RLPs, viral reads corresponding to the insert could map to homologous RLPs in the soybean genome, artificially inflating gene expression counts and impacting data normalization. Therefore BLASTN (Camacho et al., 2009) was used to compare the sequence of the B1a/B2 insert to all predicted genes in the soybean genome [Wm82.a4.v1, (Schmutz et al., 2010)]. We identified 27 genes with significant homology (E<5.1E-51, BLASTN) to either the B1a or B2 portion of the insert (**Supplementary Table 3**). Of these, 17 were considered expressed (log_2_ counts per million (cpm) >1) in our RNA-seq data. These genes were manually removed from the GFF and a new count table was generated. For each tissue, data was normalized using the Trimmed Mean of Mean (TMM) values (Robinson and Oshlack, 2010) in the Bioconductor package edgeR (Robinson and Smyth, 2007; Robinson and Smyth, 2008; Robinson et al., 2010; McCarthy et al., 2012; Zhou et al., 2014). Only genes with log_2_ cpm > 1 in at least two replicates were used in the analysis. ggplot2 (Wickham, 2009) was used to generate principal component and biological coefficient of variance plots to visually compare sample replicates to ensure reproducibility (Yin et al., 2013). Next, edgeR was used to identify genes from each tissue (leaves, stems, and roots) differentially expressed between B1a/B2 and EV plants infected with *P. gregata* and between B1a/B2 and EV plants following mock infection. In addition, to understand the difference between resistance and susceptibility in the stems, we compared gene expression between EV plants infected with *P. gregata* and mock infected (resistant) and B1a/B2 plants infected with *P. gregata* and mock infected (susceptible). Differentially expressed genes (DEGs) with a false discovery rate (FDR) < 0.01 were considered significant.

DEGs were annotated using the Gene Annotation Lookup Tool^9^ for Wm82.a4.v1. Similarly, the SoyBase GO Term Enrichment Tool^10^ for Wm82.a4 was used to identify GO terms significantly overrepresented (corrected P < 0.05) among DEG lists of interest relative to all predicted genes in the soybean genome. To visualize DEG responses across all treatment combinations, we performed hierarchical clustering of log count per million (log_2_ cpm) data in R studio^11^ using hclust (Murtagh and Legendre, 2014). Heat maps of clustered expression data were generated using Heatmap.2 in ggplot2 (Wickham, 2009).

## 3 Results

### 3.1 Silencing single clusters of receptor-like proteins

In our previous work (McCabe et al., 2018), we identified 107 RLPs in the *Rbs* region from Wm82.a2.v1. Of these, 65 were predicted to be full length, encoding LRR, B-domain, transmembrane and cytoplasmic domains. The 65 full length RLPs organized into 5 clusters (B1-B5) based on slight sequence differences in the RLP B-domain. Based on the positions of the RLP clusters and the historical *Rbs* loci, we hypothesized that RLP clusters B1 and B2 were associated with *Rbs1*, clusters B4 and B5 were associated with Rbs2, and cluster B3 was associated with *Rbs3.* We have updated the analyses to Wm82.a4.v1, identifying 120 RLPs, 76 with intact B-domains organized into five clusters (B1-B5, **Figure 1**, **Supplementary Table 1**). RLPs with partial or completely absent B-domains could not be assigned to a class and are designate as unknown (UK).

Using the updated RLP alignments, we developed six VIGS constructs targeting clusters B1 (constructs B1a and B1b), B2, B3, B4 and B5 (**Supplementary Table 2**). Primers were designed to span conserved regions within each aligned cluster. The B1a, B1b and B2 constructs were developed using L78-4094 (*Rbs1*) as the template DNA. The B3 construct was developed using PI 437970 (*Rbs3*) as the template DNA. The B4 and B5 constructs were developed from PI 437833 (*Rbs2*) DNA. Based on BLASTN (E<0.01) analyses (Camacho et al., 2009), the B1a, B1b and B2, B3, B4 and B5 constructs each targeted 18, 4, 8, 16, 44 and 16 RLPs, respectively, relative to the Wm82.a4 genome sequence (**Supplementary Table 3**). Across all six constructs, we identified 18 identified RLP targets outside the *Rbs* loci, however eight were located elsewhere on chromosome 16 and 5 were unassigned to a chromosome. Sequence alignments for each of the single gene constructs and their BLAST targets in Williams82.a4.v1 are provided in **Supplementary Figures 1–6**. Constructs B1a, B1b and B2, were tested on L78-4094 (*Rbs1*), construct B3 was tested on PI 437970 (*Rbs3*), and constructs B4 and B5 were tested on PI 437833 (*Rbs2).* Constructs were tested with *P. gregata* infection and mock infection. While plants treated with a VIGS vector often appeared smaller than mock VIGS plants, no loss of resistance was observed, and the susceptible check behaved as expected (data not shown). Repeated testing failed to identify a silencing construct resulting in loss of resistance to *P. gregata*.

### 3.2 Silencing multiple clusters of receptor-like proteins

**Figure 2:**
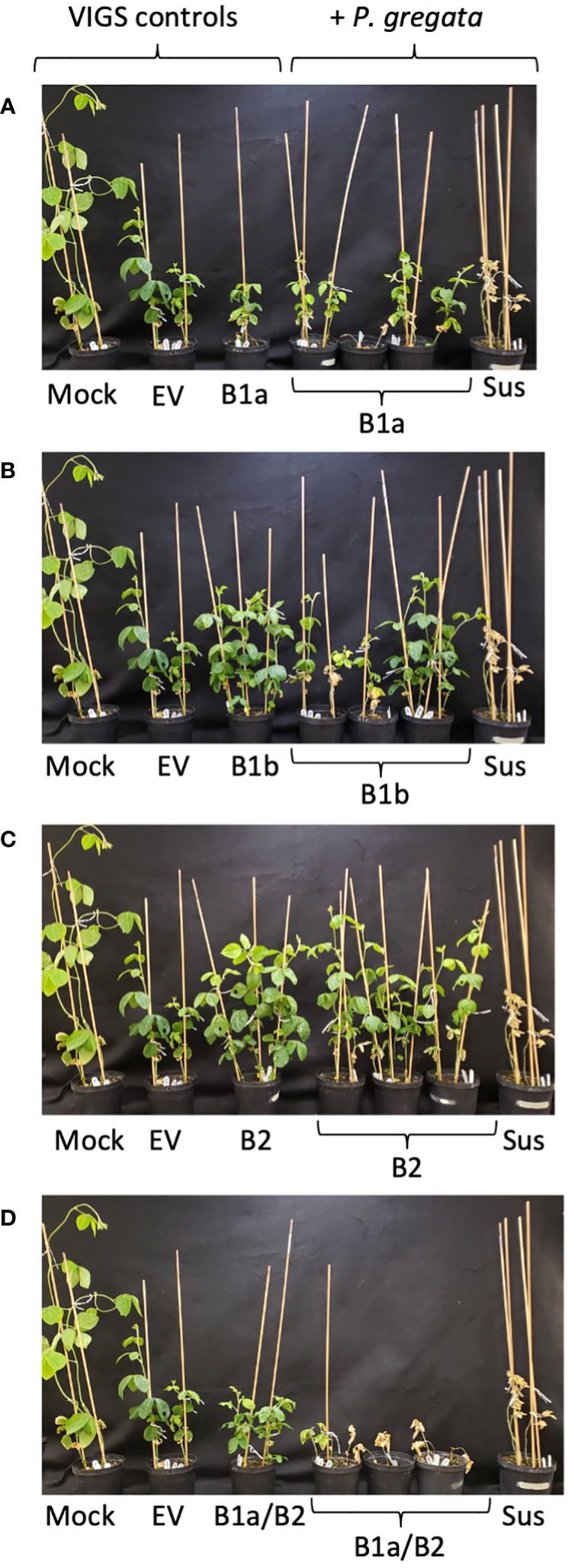
The B1a/B2 VIGS construct silences resistance to *P. gregata* in L78-4094 (*Rbs1*). **(A–C)** VIGS constructs developed for individual RLP clusters B1a, B1b and B2, do not silence resistance to *P. gregata*. **(D)** VIGS construct B1a/B2, targeting two RLP clusters, results in loss of resistance to *P. gregata* in L78-4094. Controls for VIGS include Mock VIGS, empty vector (EV), and VIGS infected but not infected with *P. gregata*. Controls for infection with *P. gregata* include the susceptible check Corsoy79 (Sus).

Three scenarios could explain the lack of phenotype found above. In the first scenario, while VIGS constructs were designed from conserved regions in the cluster alignment, outlier genes might not be adequately target by the construct. Second, it’s possible the resistance genes present in the resistant PI(s) are significantly different from those found in Williams82 reference genome. Without additional genomic sequences from resistant PI, this scenario cannot be tested. In the final scenario, multiple genes, representing distinct clusters, confer resistance. Only by silencing multiple clusters can loss of resistance be detected. To test this, we took advantage of the Gibson Assembly Method (Casini et al., 2015) to ligate PCR products representing different clusters into a single VIGS construct. We created constructs B1a/B2 and B1b/B2 VIGS to silence *Rbs1* and construct B4/B5 to silence *Rbs2* (**Supplementary Table 2**). BLASTN (E<0.01) analyses against the Wm82.a4 primary transcripts suggest the B1a/B2, B1b/B2 and B4/B5 constructs could target 27, 12 and 60 RLPs, respectively. Across all three constructs, we identified 17 RLP targets outside the *Rbs* loci, however eight were located elsewhere on chromosome 16 and 6 were unassigned to a chromosome. No loss of resistance was observed with the B1b/B2 construct in L78-4094 (*Rbs1*) plants or the B4/B5 constructs in PI 437833 (*Rbs2*), data not shown. However, in three replicate tests, L78-4094 (*Rbs1*) plants infected with the B1a/B2 BPMV VIGS construct had a complete loss of BSR resistance 5 weeks after infection (**Figure 2**).

It is worth noting the B1a/B2 construct would have silenced all three gene candidates identified by Rincker et al. (Rincker et al., 2016a; Rincker et al., 2016b). The B1a portion of the B1a/B2 construct showed 98% nucleotide identity with Glyma.16g169900 and the B2 portion showed 95% and 97% nucleotide identity with Glyma.16g169600 and Glyma.16g169700, respectively. However, our results demonstrate that two RLP clusters and at least two RLPs are responsible for *Rbs1*-mediated resistance to *P. gregata.* To determine if the historical genotypes thought to contain different *Rbs* genes were a single *Rbs* locus, we also tested the B1a/B2 construct in PI 437833 (*Rbs2*) and PI 437970 (*Rbs3*). No loss of resistance was observed (data not shown), confirming these lines carry additional *P. gregata* resistance genes. This supports the model by Bachman and Nickell (Bachman and Nickell, 2000), suggesting multiple genes are required for resistance to *P. gregata.*

### 3.3 RNA-seq analysis to identify genes responding to B1a/B2 silencing

To understand how RLPs confer resistance to *P. gregata*, we conducted RNA-seq analyses of B1a/B2 and EV plants nine days after silencing and seven days after infection with *P. gregata* or mock infection. While plants were harvested before symptom development, our previous studies suggest RNA isolated later than 1 week after *P. gregata* infection from susceptible plants was degraded and not suitable for RNA-seq analyses (McCabe et al., 2018).

FASTQ Screen (Wingett and Andrews, 2018) was used to screen sequence data from 48 RNA-seq libraries. Of these, 22 had VIGS infection rates less than 4% and were removed from further analyses. The remaining 26 libraries were mapped to the soybean reference genome, Wm82.a4 (Schmutz et al., 2010). Of the initial 754,211,277 150 bp single end reads, 279, 429,128 were successfully mapped (**Supplementary Table 4**). The percentage of reads corresponding to BPMV in each library varied from 4 to 87.5%. These sequences are available from the National Center for Biotechnology Sequence Read Archive, BioProject Accession PRJNA1014479.

**Figure 3:**
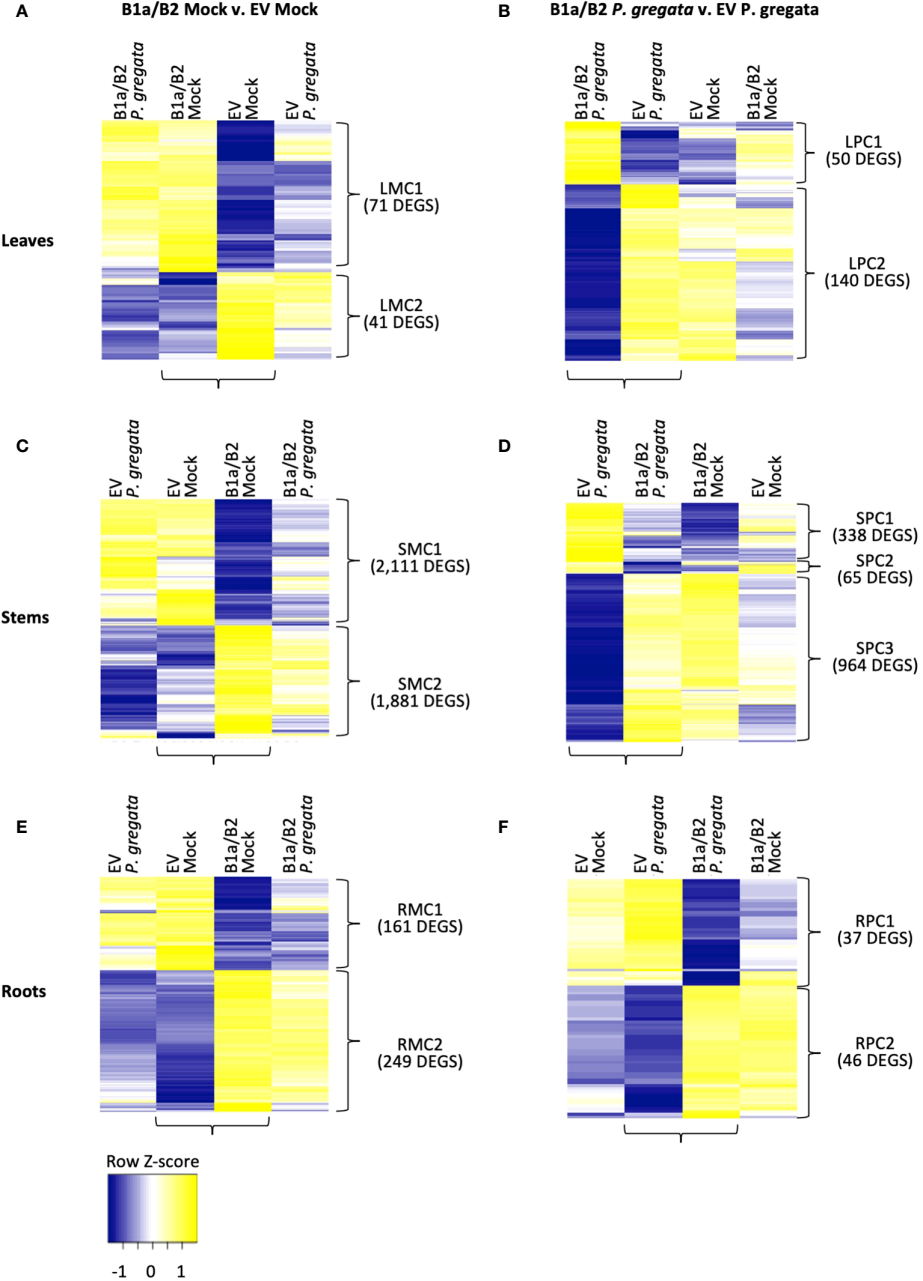
DEGs responding to silencing (B1a/B2 – EV) in mock infected **(A, C, E)** and *P. gregata* infected **(B, D, F)** leaves, stems and roots. DEGs were identified by comparing the samples indicated by horizontal brackets. Additional samples are provided to examine response to pathogen infection **(A, C, E)** or mock infection **(B, D, F)** in DEGs of interest. Vertical brackets indicate clusters used for GO enrichment. Hierarchical clustering of log_2_ cpm data was performed to characterize expression changes across samples, with Z-scores represented in the heatmaps above.

Using a false discovery rate (FDR) < 0.01, we identified differentially expressed genes (DEGs) between B1a/B2 and EV silenced plants infected with *P. gregata* and mock infected B1a/B2 and EV silenced plants (**Supplementary Tables 5**–**10**). This allowed us to directly identify genes whose expression pattern changed in response to silencing of the candidate RLPs in two different conditions and in three different tissues, generating six DEG list of interest. To explore the DEGs at a global level, we performed hierarchical clustering of log_2_ cpm expression data of DEGs within a tissue and treatment using all the data from EV and B1a/B2 plants infected with *P. gregata* or mock infected. The resulting heat maps (**Figure 3**) help to identify genes impacted by B1a/B2 silencing that would also normally respond to *P. gregata* infection. Within each heat map, we identified clusters of DEGs with similar expression patterns across samples. We used the SoyBase GO Enrichment Tool^12^ to assign significantly overrepresented (Corrected P-value < 0.05) gene ontology (GO) biological process terms to a cluster, relative to all predicted genes in the reference genome. Finally, to understand the difference between resistance and susceptibility, we used the stem data to compare *P. gregata* and mock-infected plants in EV and B1a/B2 plants.

In the leaves, we identified 112 DEGs when comparing B1a/B2 to EV plants under mock conditions (**Figure 3A**). Leaf mock cluster 1 (LMC1), had no significant GO terms associated with it, however LMC2 was associated with response to brassinosteroids (GO:0009741), cell wall biogenesis (GO:0042546) and xyloglucan metabolism (GO:0010411). When comparing leaves of *P. gregata* infected B1a/B2 and EV plants, we identified 190 DEGs (**Figure 3B**). Leaf pathogen-infected cluster 1 (LPC1) was associated with amino acid related functions such as asparagine biosynthesis (GO:0070981 and GO:0006529), glutamine metabolism (GO:0006541) and amino acid catabolism (GO:0009063). LPC2 was associated with cell wall biogenesis (GO:0042546) and xyloglucan metabolism (GO:0010411), like LMC2, and also with proteolysis (GO:0006508) and regulation of protein serine/threonine phosphatases (GO:0080163). Interestingly, when we examine the DEGs from mock infected leaves (**Figure 3A**), we see little variation between mock infection and *P. gregata* infection with a silencing construct. However, for DEGs identified by comparing constructs under *P. gregata* infection (**Figure 3B**), DEGs were dramatically differentially expressed relative to the mock controls.

In the stems, we identified 3,992 DEGs when comparing B1a/B2 to EV plants under mock conditions (**Figure 3C**). While the DEGS are distinct, expression patterns mirror those observed for mock infected leaves. Stem mock cluster 1 (SMC1) was strongly repressed in B1a/B2 plants, relative to EV. SMC1 was associated with defense [response to chitin (GO:0010200), defense response to fungus (GO:0050832), and defense response to virus by host (GO:0050691)], stress tolerance [response to hypoxia (GO:0071456), heat (GO:0034605) and protein folding (GO:0006457)], response to unfolded protein (GO:0006986 and GO:0034620), chaperone cofactor-dependent protein refolding (GO:0051085). GO terms associated with protein folding are also associated with stress and defense responses. In plants, the unfolded protein response (UPR) is vital for host defenses against viral, bacterial and fungal pathogens (Verchot and Pajerowska-Mukhtar, 2021). Signaling genes repressed by B1a/B2 silencing included 18 homologs of AtWRKY transcription factors including AtWRKY33, AtWRKY40, AtWRKY50, At WRKY51 and AtWRKY70. WRKY transcription factors are important regulators of pathogen-associated molecular pattern (PAMP) triggered immunity (PTI), effector triggered immunity, phytohormones such as jasmonic and salicylic acid (JA and SA), and abiotic stress responses (Chen et al., 2019). A homolog of *AtIOS1*, a malectin-like LRR RLK required for PTI, was also repressed by B1a/B2 silencing. Perhaps most interesting, B1a/B2 silencing repressed the expression of ten chromosome 16 RLPs unrelated to the B1a and B2 clusters. This includes four RLPs we were unable to assign to an RLP class, three B3 RLPs and three B4 RLPs. DEGs in SMC2 were induced in B1a/B2 silenced plants relative to EV following *P. gregata* infection or mock infection. Significant SMC2 GO terms were associated with the cell wall (cell wall biogenesis (GO:0009832, GO:0009833 and GO:0009834), regulation of cell wall biogenesis (GO:2000652)), cellulose biosynthesis (GO:0030244) and catabolism (GO:0030245), lignin biosynthesis (GO:0009809) and catabolism (GO:0046274), xylan biosynthesis (GO:0045492) and rhamnogalacturonan I side chain metabolism (GO:0010400), iron homeostasis (GO:0055072) and response to response to cyclopentenone (GO:0010583).

Comparing B1a/B2 to EV stems following *P. gregata* infection identified 1,367 DEGS divided into three clusters (**Figure 3D**). Stem pathogen cluster 1 (SPC1) was induced by pathogen infection in EV plants but repressed in B1a/B2 plants. SPC1 was associated with two significantly overrepresented GO terms: response to herbivore (GO:0080027) and chitin catabolism (GO:0006032). Response to herbivore included three homologs of pathogenesis related (PR) protein 4 (*AtPR4*) and two homologs of *AtPR1B*. Chitin catabolism included three homologs of *AtPR3*, three homologs *AtLYS1*, and two homologs of *AtCHIV* (all chitinases), two homologs of *ATCTL2* (chitinase-like), and *AtCHIC*, a glycosyl hydrolase with a chitinase domain. AtLYS1 activity releases peptidoglycans from bacterial cell walls, triggering plant immune responses (Liu et al., 2014). SPC2 had no significant GO terms. SPC3 GO terms mirrored SMC2, with ten GO terms related to the cell wall and one GO term related to iron homeostasis common to both groups of DEGs. It is worth noting that 739 DEGs were common between stem DEGS mock infected or infected with *P. gregata*. Unlike the DEGs identified from *P. gregata* infected leaves, that gain pathogen responsiveness following B1a/B2 silencing, DEGs from stems were expressed similarly following infection and mock infection in B1a/B2 silenced plants.

We identified 410 DEGs from roots between EV and B1a/B2 mock treated plants (**Figure 3E**). In root mock cluster 1 (RMC1), only the GO term response to symbiotic fungus (GO:0009610) was significant. RMC2 had three significant GO terms including membrane disassembly (GO:0030397), lipid oxidation (GO:0034440) and oxylipin biosynthesis (GO:0031408). We identified 83 DEGs between EV and B1a/B2 plants infected with *P. gregata* (**Figure 3F**). Root pathogen cluster 1 (RPC1) had no significant GO terms while for RPC2, xylan catabolism (GO:0045493) was the only significant GO term.

### 3.4 RNA-seq analysis to identify genes responding *P. gregata* in empty vector and B1a/B2 silenced plants

**Figure 4:**
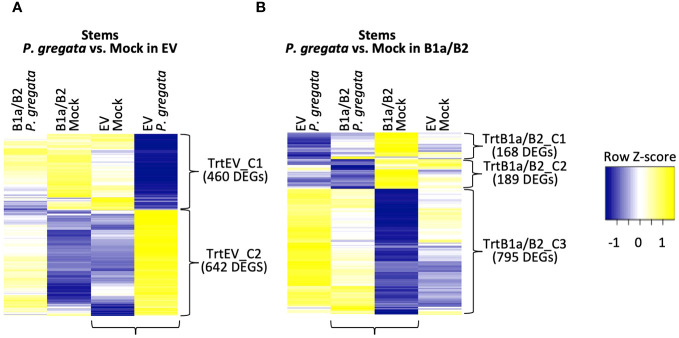
DEGs responding to *P. gregata* infection in EV **(A)** and B1a/B2 **(B)** stems. DEGs were identified by comparing the samples indicated by horizontal brackets. Additional samples are provided to examine response in the alternate VIGS construct. Vertical brackets indicate clusters used for GO enrichment. Hierarchical clustering of log_2_ cpm data was performed to characterize expression changes across samples, with Z-scores represented in the heatmaps.

Based on these analyses, it appeared that the B1a/B2 construct had the most impact in the stems, as did *P. gregata* infection. To understand how the DEGs described above impacted disease outcome, we also need to see how EV and B1a/B2 plants responded to infection. Therefore, we compared *P. gregata* infected and mock infected plants corresponding to each construct, focusing specifically on the stems. We identified 1,102 and 1,152 DEGs responding to infection in stems from EV and B1a/B2 plants, respectively (**Supplementary Tables 12**, **13**). Of these, 363 DEGs were in common. As described above, DEGs were identified by comparing two groups of interest, but we were also interested in how the DEGs were expressed in the other samples. Heatmaps for stem DEGs responding to *P. gregata* infection in EV and B1a/B2 plants are shown in **Figure 4**.

For *P. gregata* response in EV, we identified two clusters: TrtEV_C1 and TrtEV_C2. GO terms significant in TrtEV_C1 include many of the cell wall GO terms previously described including cell wall biogenesis (GO:0009832, GO:0009833 and GO:0009834), cellulose biosynthesis (GO:0030244), xylan biosynthesis (GO:0045492) and rhamnogalacturonan I side chain metabolism (GO:0010400). While these DEGs were repressed by *P. gregata* in EV (TrtEV_C1, **Figure 4A**), they were induced in B1a/B2 mock plants relative to EV mock (SMC2, **Figure 3C**) and in B1a/B2 *P. gregata* infected plants relative to EV infected plants (SPC3, **Figure 3D**). This suggests B1a/B2 silencing, silences genes involved in cell wall pathways. GO terms significant in TrtEV_C2 include many GO terms associated with defense and stress tolerance including chalcone biosynthesis (GO:0009715), response to gravity (GO:0009629), UV-B (GO:0010224), and karrikin (GO:0080167), regulation of anthocyanin (GO:0031540) ethylene biosynthesis (GO:0010365), chitin (GO:0006032) and toxin catabolism (GO:0009407), defense response to fungus (incompatible, GO:0009817) and flavonoid biosynthesis (GO:0009813). These DEGs were strongly induced in response to *P. gregata* in EV plants, but only weakly induced in B1a/B2 plants (**Figure 4A**). Of these GO terms, only chitin catabolism (GO:0006032) was found in other stem clusters (SPC1, **Figure 3D**). Cluster SPC1, was strongly induced in EV plants relative to B1a/B2 plants, both infected with *P. gregata*.

For *P. gregata* response in B1a/B2 stems, we identified three clusters. TrtB1a/B2_C1 was significantly overrepresented with GO terms inositol phosphorylation (GO:0052746) and inositol triphosphate metabolism (GO:0032957). This cluster was weakly repressed in response to infection in B1a/B2 stems, but strongly repressed in response to infection in EV stems. TrtB1a/B2_C2 was significantly overrepresented with GO terms mitochondrial transport (GO:0006839), glycerol ether metabolism (GO:0006662), and syncytium formation (GO:0006949). This cluster was strongly repressed in response to infection in B1a/B2 stems, but weakly repressed in EV stems. TrtB1a/B2_C3 was significantly overrepresented with regulation of defense response to virus (GO:0050691) and regulation of defense response (GO:0031347), response to unfolded protein GO:0006986), bacterium (GO:0009617), salicylic acid (GO:0009751) and chitin (GO:0010200), defense response to fungus (GO:0050832), chitin catabolism (GO:0006032) and hormone-mediated signaling pathway (GO:0009755). This cluster was weakly induced in response to infection in B1a/B2 stems, but strongly induced in EV stems. GO terms regulation of defense response to fungus (GO:0050832), response to chitin (GO:0010200) and response to folded protein (GO:0006986) were also identified as significant in SMC1 (**Figure 3C**). While largely induced in EV stems regardless of pathogen infection, they were repressed in B1a/B2 stems.

## 4 Discussion

Allelism studies have been used to identify BSR resistance genes *Rbs1, Rbs2*, and *Rbs3* (Sebastian and Nickell, 1985; Hanson et al., 1988; Willmont and Nickell, 1989). While these studies suggest the three genes are tightly linked, but distinct loci, GWA and fine-mapping studies suggest they could be alleles of a single locus (Rincker et al., 2016a; Rincker et al., 2016). Other studies on the inheritance of BSR resistance suggest the presence of modifier genes (Eathington et al., 1995; Lewers et al., 1999) or even oligogenic inheritance (Bachman and Nickell, 2000), where multiple genes are required for BSR resistance. Interpreting the results of these studies is further complicated by differences in *P. gregata* inoculation methods (stem and root), study conditions (growth chamber or greenhouse) and the type of and number of molecular markers used in the study. To introduce BSR resistance into elite germplasm, we need a clear understanding of the *Rbs* locus.

In our previous work (McCabe et al., 2018), we leveraged mapping data and bioinformatics to identify and characterize 100+ RLPs located within the *Rbs* loci. The *Rbs* RLPs were homologous to apple scab resistance genes (*HcrVf*) and tomato *Cladosporium fulvum* (*Cf*), Verticillium wilt (*Ve*), and *Trichoderma viride* resistance genes (*LeEIX*). Further, the *Rbs* RLPs had conserved cysteine pairs typical of resistance RLPs (Fritz-Laylin et al., 2005) and were clustered within the genome. Based on the location of these clusters, we hypothesized that different RLP clusters would correspond to different *Rbs* genes. Here, we developed VIGS constructs for each RLP cluster (B1a, B1b, B2, B3, B4 and B5). We then tested each construct for its ability to silence *P. gregata* resistance in the predicted genetic background. None of the single cluster VIGS constructs resulted in a loss of resistance phenotype. Since previous studies have suggested the presence of modifiers (Eathington et al., 1995; Lewers et al., 1999) or even oligogenic inheritance (Bachman and Nickell, 2000), we then developed VIGS constructs that could target pairs of RLP clusters. Construct B1a/B2 silenced *P. gregata* resistance in L78-4094 (*Rbs1*), demonstrating at least two genes, from two clusters, are required for *Rbs1*-mediated resistance. This supports the oligogenic model proposed by Bachman and Nickell (Bachman and Nickell, 2000). Since construct B1a/B2 was unable to silence *P. gregata* resistance in either PI 437833 (*Rbs2*) or PI 437970 (*Rbs3*), it suggests this region on chromosome 16 contains additional *P. gregata* resistance genes.

Based on BLAST (Camacho et al., 2009) analyses, we predicted that construct B1a/B2 could target as many as 27 different RLPs. To narrow the number of candidate genes for *Rbs1*, we examined the original count tables, prior to RLP removal, for roots and stems generated in our RNA-seq pipeline. We focused on the EV libraries, where no inflation of RLP counts (due to viral reads) would have occurred. Assuming the *Rbs1* resistance genes would be expressed in all EV samples (at least one read per sample) and would be expressed in roots and stems, narrowed the candidates for B1a to three genes (Glyma.16G170700, Glyma.16G171500 and Glyma.16G172300) and for B2 to two genes (Glyma.16G169200 and Glyma.16G169500). This demonstrates the utility of coupling VIGS with RNA-seq to characterize disease resistance loci.

In addition to understanding what gene(s) condition resistance to *P. gregata*, we were also interested in downstream disease resistance signaling. Typically, when conducting experiments with resistant and susceptible genotypes, our group has focused on response to infection by comparing pathogen inoculated plants to mock inoculated plants. However, when VIGS is incorporated, measuring a lack of response becomes more difficult. Therefore, in this study we compared B1a/B2 plants to EV plants in either *P. gregata* infected tissue or mock infected tissue. This allowed us to identify all genes whose expression changed in response to silencing. This revealed the largest response was in the stems, therefore we also used the stem data to examine the response to infection in B1a/B2 and EV plants to put the genes in context.

At a global level, B1a/B2 silencing induced DEGs involved in cell wall biogenesis, lipid oxidation, the unfolded protein response and iron homeostasis. While these pathways were largely identified in the stems, we also detected similar evidence in roots and leaves. In plants, changes to the structural integrity of the cell wall activate the conversion of fatty acids in the phospholipid layer to lipidhydroperoxides (Spiteller, 2003; Singh et al., 2022). If damage is significant, peroxyl radicals generated by lipidhydroperoxides will attack proteins and other biological molecules, resulting in programed cell death and the release or iron ions. In addition, lipidhydroperoxides, phospholipases and desaturases help regulate the lipoxygenase pathway, which generates jasmonic acid (JA) precursors. Crosstalk between JA and other plant hormones balance growth with stress and defense responses (Yang et al., 2019). JA is induced in response to necrotrophic fungi in both resistant and susceptible interactions (Macioszek et al., 2023). In EV plants, these pathways were strongly repressed in response to *P. gregata* infection in the stem [**Figure 3D** (SPC3), **Figure 4A** (TrtEV_C1), **Supplementary Tables 11**, **14**]. In contrast, they were induced in stems of B1a/B2 silenced plants, regardless of pathogen infection. This suggests that repression of these pathways in response to *P. gregata* infection is an important defense mediated by the *Rbs* RLPs.

In contrast, B1a/B2 silenced plants repressed expression of numerous genes involved in defense and defense signaling. Defense genes repressed by the B1a/B2 silencing included 18 homologs of AtWRKY transcription factors including AtWRKY33, AtWRKY40, AtWRKY50, At WRKY51 and AtWRKY70. WRKY transcription factors are important regulators of defense and immune pathways. For example, AtWRKY33 is essential for salicylic acid (SA) mediated defenses against the necrotrophic fungus *Botrytis cinerea* (Birkenbihl et al., 2012). AtWRKY40 (Schön et al., 2013) and AtWRKY70 (Eulgem and Somssich, 2007) regulate SA and jasmonic acid mediated defense pathways. AtWRKY50 and AtWRKY51 mediate salicylic acid dependent repression of JA defense signaling (Gao et al., 2011). In addition, B1a/B2 silencing repressed ten chromosome 16 RLPs unrelated to the B1a and B2 clusters.

Given these results, we were also interested in determining if RLPs elsewhere in the genome could have disease resistance functions. Recently, Restrepo-Montoya et al. (2020) used a computational approach to identify all RLPs and RLKs present in the genomes of selected legumes. They identified 468 RLPs from soybean (Wm82.a2.v1). Using the regular expression pattern C\w{1},CC\w{1,8}C, we searched the protein sequences of the 468 RLPs for the conserved four cysteine motif identified by Fritz-Laylin et al. (2005) that distinguished between RLPs involved in development and defense across multiple species. Of the 468 RLPs identified by Restrepo-Montoya et al. (2020), 118 contained the conserved cysteine motif (data not shown). Of these, 61 corresponded to the *Rbs* loci on chromosome 16, while the remaining 57 were found on all other chromosomes except chromosome 2. The largest cluster, found on chromosome 10, contained five RLPs. These finding suggest that the *Rbs* locus on chromosome 16 may be the largest hotspot for fungal disease resistance in the soybean genome. This region has also been associated with resistance to frog eye leaf spot (*Rcs3*), soybean cyst nematode, sudden death syndrome and soybean mosaic virus (**Supplementary Figure 7**). McDonald et al. (2023) recently fine-mapped *Rcs3* to Gm16:32,722,648 to 34,426,760 (updated to Wm82.a4.v1). This region corresponds to RLP clusters 1, 2 and 3. It is possible that the VIGS constructs developed here could be a useful tool for characterizing resistance to these other pathogens.

In summary, silencing of two RLP clusters resulted in the loss of *Rbs1*-mediated resistance to *P. gregata*, supporting the oligogenic inheritance model suggested by Bachman and Nickell (2000). Other RLP genes within the *Rbs* loci, also likely impact *P. gregata* resistance. Better understanding BSR resistance mechanisms will enable faster identification of novel resistant germplasm and easier integration of resistance into elite soybean germplasm. These findings highlight the importance of chromosome 16 RLPs and present new avenues for future studies on BSR resistance and resistance to other fungal soybean diseases.

## Data availability statement

The data presented in this study are deposited in the NCBI SRA database, accession number PRJNA1014479.

## Author contributions

CM: Conceptualization, Data curation, Formal analysis, Funding acquisition, Investigation, Methodology, Project administration, Supervision, Writing – original draft, Writing – review & editing. LL: Conceptualization, Data curation, Formal analysis, Investigation, Methodology, Writing – original draft, Writing – review & editing. JO: Conceptualization, Methodology, Writing – review & editing. MG: Conceptualization, Data curation, Formal analysis, Funding acquisition, Investigation, Methodology, Project administration, Supervision, Writing – original draft, Writing – review & editing.

## Footnotes

1 SoyBase Gene Annotation Tool. https://www.soybase.org/genomeannotation/.

2 FastQC: a quality control tool for high throughput sequence data. http://www.bioinformatics.babraham.ac.uk/projects/fastqc/.

3 Scythe- a Bayesian adapter trimmer. https://github.com/vsbuffalo/scythe

4 FASTX-Tookit. http://hannonlab.cshl.edu/fastx_toolkit/.

5 Sickle- a windowed adaptive trimming tool for FASTQ files using quality. https://github.com/najoshi/sickle/.

6 R Core Team. R: A language and environment for statistical computing. R Foundation for Statistical Computing, Vienna, Austria. https://www.R-project.org/.

7 Rsamtools: Binary alignment (BAM), FASTA, variant call (BCF), and tabix file import. R package version 2.16.0, https://bioconductor.org/packages/Rsamtools.

8 Counting reads with summerizeOverlaps. http://bioconductor.org/packages/devel/bioc/vignettes/GenomicAlignments/inst/doc/summarizeOverlaps.pdf.

9 SoyBase Gene Annotation Tool. https://www.soybase.org/genomeannotation/.

10 SoyBase GO Term Enrichment Tool. https://www.soybase.org/goslimgraphic_v2/dashboard.php.

11 RStudio: integrated development for R. http://www.rstudio.com/.

12 SoyBase GO Term Enrichment Tool. https://www.soybase.org/goslimgraphic_v2/dashboard.php.
