# Supplementary material for: Virus induced gene silencing confirms oligogenic inheritance of brown stem rot resistance in soybean
Source: Front Plant Sci. 2024 Jan 8;14:1292605. doi: 10.3389/fpls.2023.1292605 (PMC10801082; doi:10.3389/fpls.2023.1292605)
Supplement: Supplementary file 2 [file Presentation_1.pptx]

## Slide 1
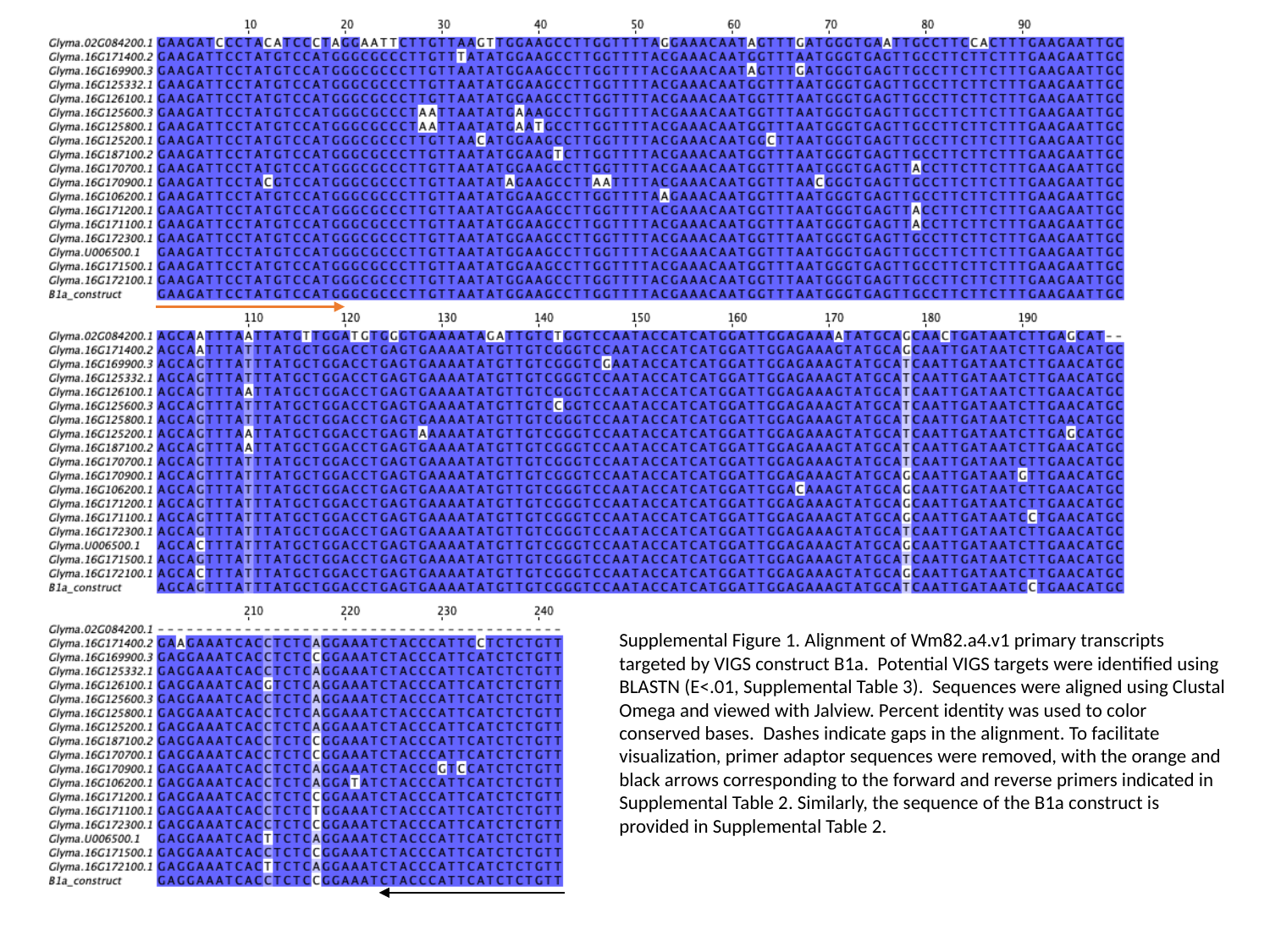

Supplemental Figure 1. Alignment of Wm82.a4.v1 primary transcripts targeted by VIGS construct B1a. Potential VIGS targets were identified using BLASTN (E<.01, Supplemental Table 3). Sequences were aligned using Clustal Omega and viewed with Jalview. Percent identity was used to color conserved bases. Dashes indicate gaps in the alignment. To facilitate visualization, primer adaptor sequences were removed, with the orange and black arrows corresponding to the forward and reverse primers indicated in Supplemental Table 2. Similarly, the sequence of the B1a construct is provided in Supplemental Table 2.

## Slide 2
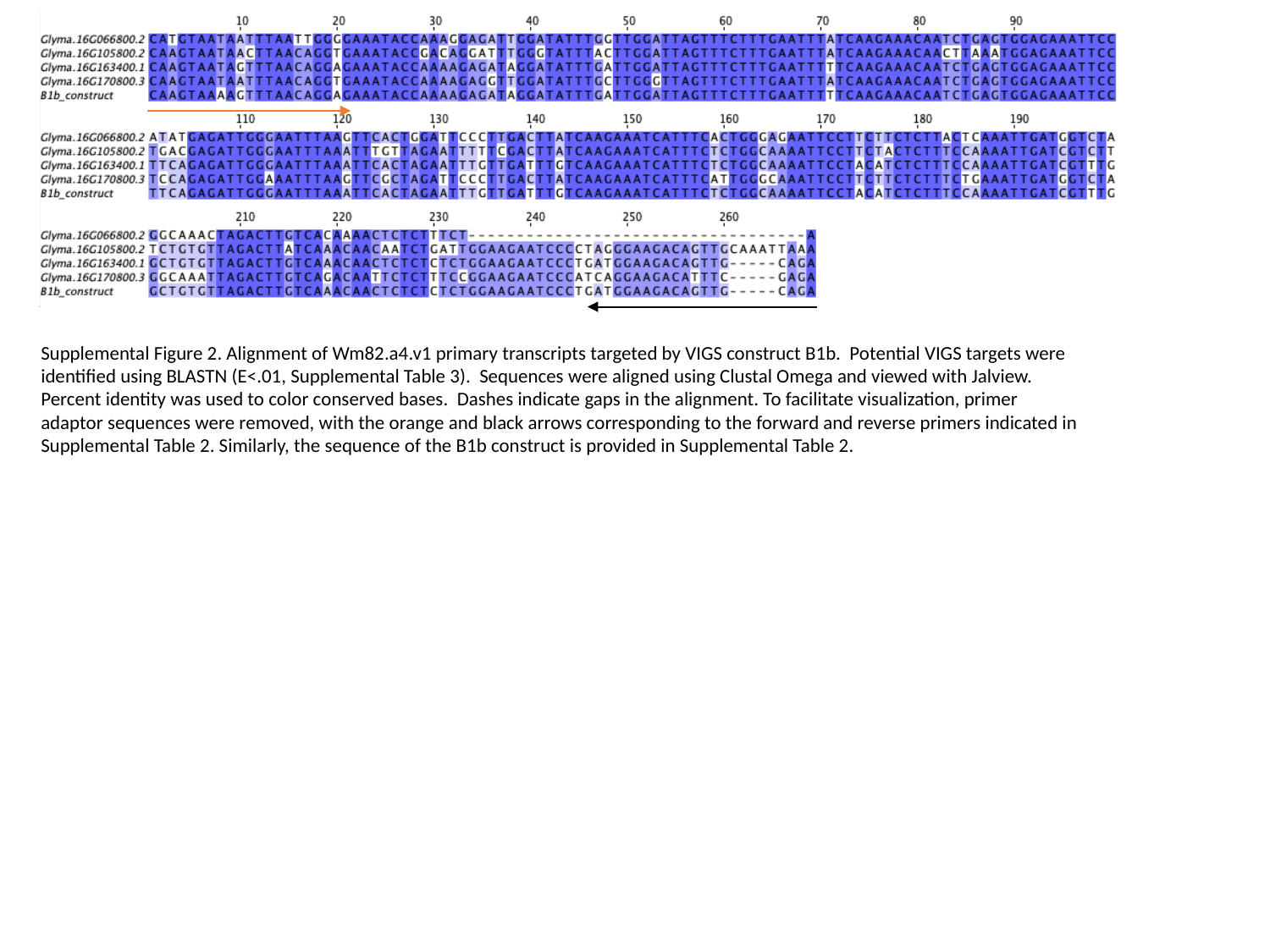

Supplemental Figure 2. Alignment of Wm82.a4.v1 primary transcripts targeted by VIGS construct B1b. Potential VIGS targets were identified using BLASTN (E<.01, Supplemental Table 3). Sequences were aligned using Clustal Omega and viewed with Jalview. Percent identity was used to color conserved bases. Dashes indicate gaps in the alignment. To facilitate visualization, primer adaptor sequences were removed, with the orange and black arrows corresponding to the forward and reverse primers indicated in Supplemental Table 2. Similarly, the sequence of the B1b construct is provided in Supplemental Table 2.

## Slide 3
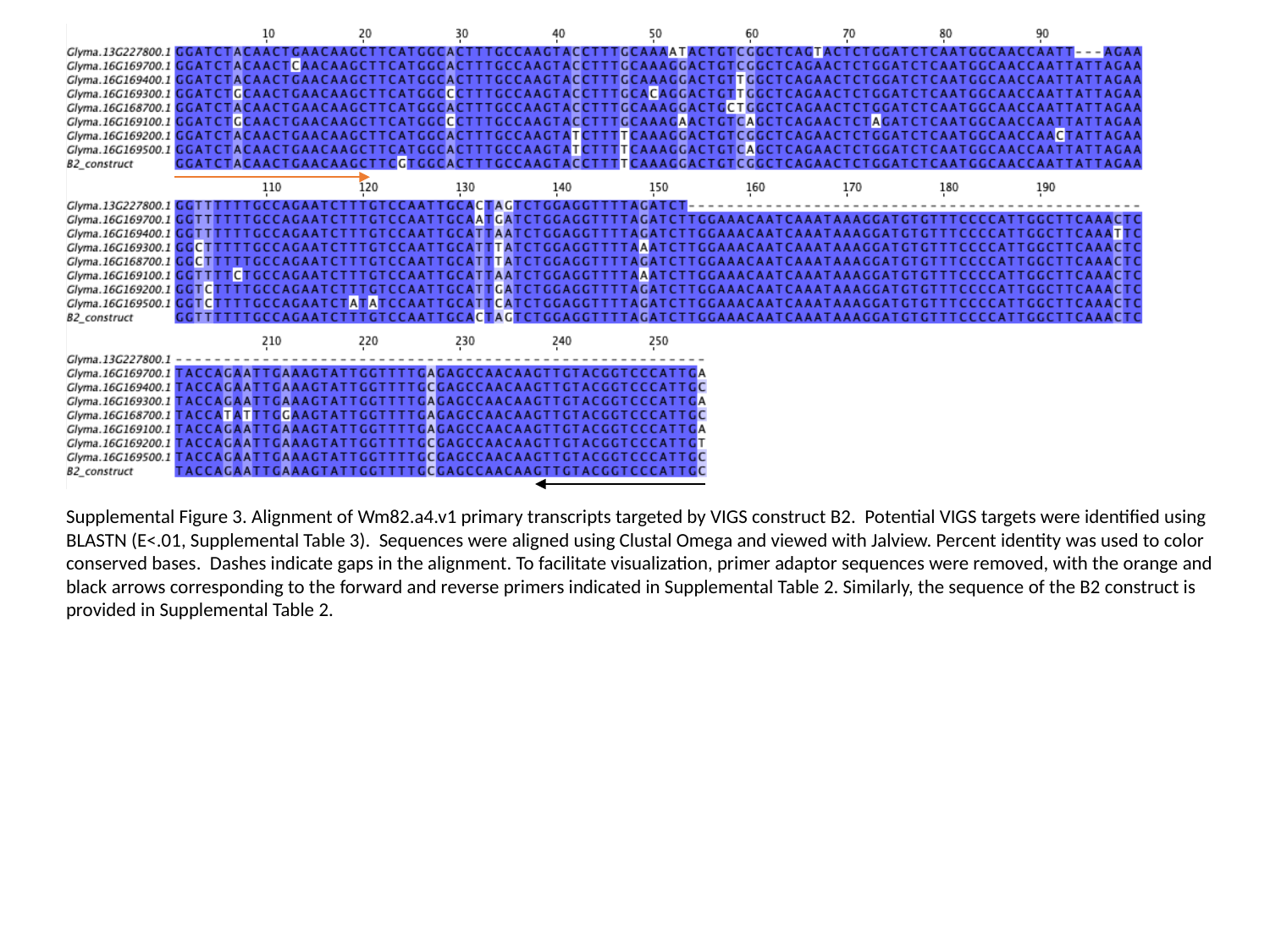

Supplemental Figure 3. Alignment of Wm82.a4.v1 primary transcripts targeted by VIGS construct B2. Potential VIGS targets were identified using BLASTN (E<.01, Supplemental Table 3). Sequences were aligned using Clustal Omega and viewed with Jalview. Percent identity was used to color conserved bases. Dashes indicate gaps in the alignment. To facilitate visualization, primer adaptor sequences were removed, with the orange and black arrows corresponding to the forward and reverse primers indicated in Supplemental Table 2. Similarly, the sequence of the B2 construct is provided in Supplemental Table 2.

## Slide 4
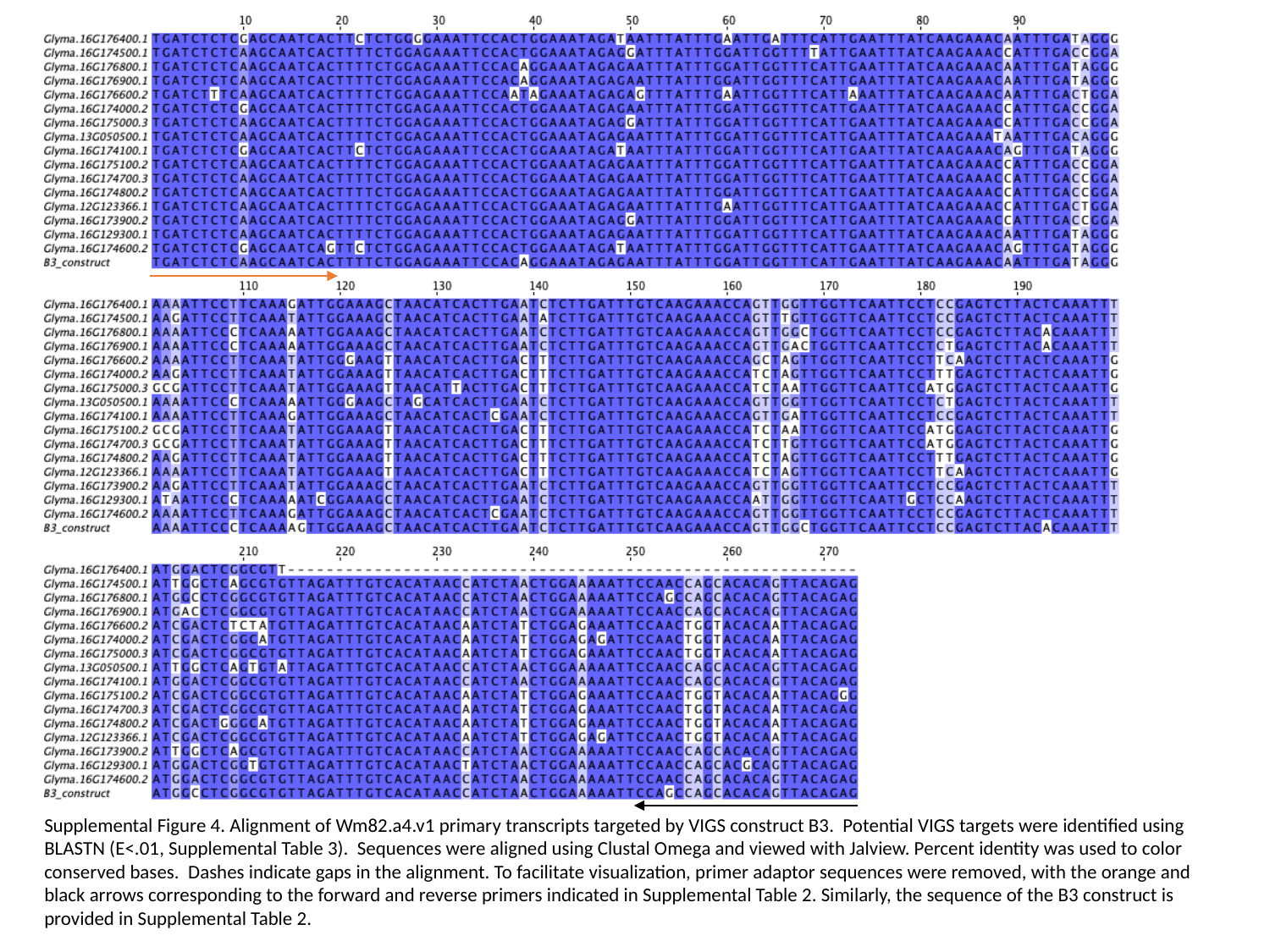

Supplemental Figure 4. Alignment of Wm82.a4.v1 primary transcripts targeted by VIGS construct B3. Potential VIGS targets were identified using BLASTN (E<.01, Supplemental Table 3). Sequences were aligned using Clustal Omega and viewed with Jalview. Percent identity was used to color conserved bases. Dashes indicate gaps in the alignment. To facilitate visualization, primer adaptor sequences were removed, with the orange and black arrows corresponding to the forward and reverse primers indicated in Supplemental Table 2. Similarly, the sequence of the B3 construct is provided in Supplemental Table 2.

## Slide 5
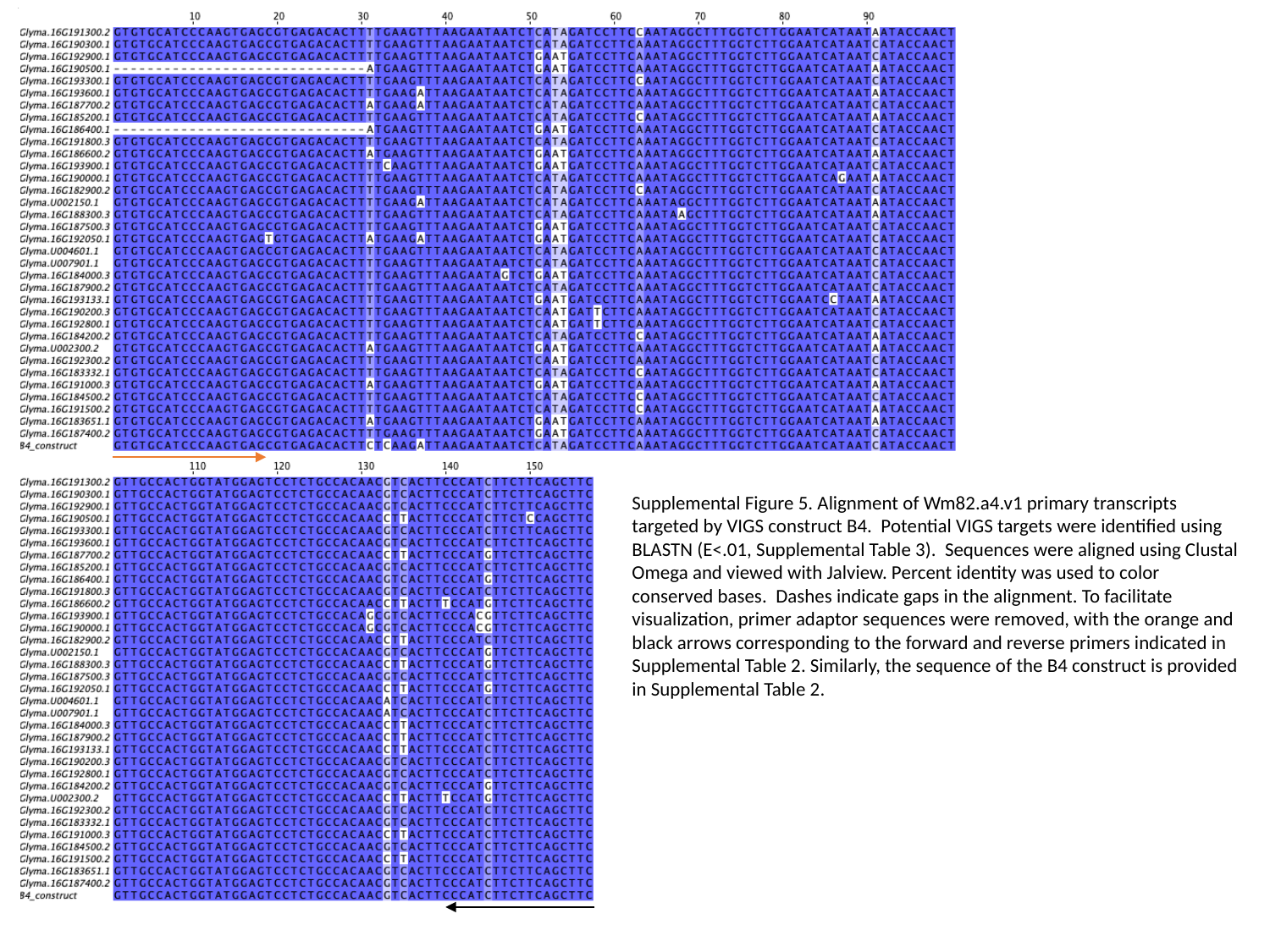

Supplemental Figure 5. Alignment of Wm82.a4.v1 primary transcripts targeted by VIGS construct B4. Potential VIGS targets were identified using BLASTN (E<.01, Supplemental Table 3). Sequences were aligned using Clustal Omega and viewed with Jalview. Percent identity was used to color conserved bases. Dashes indicate gaps in the alignment. To facilitate visualization, primer adaptor sequences were removed, with the orange and black arrows corresponding to the forward and reverse primers indicated in Supplemental Table 2. Similarly, the sequence of the B4 construct is provided in Supplemental Table 2.

## Slide 6
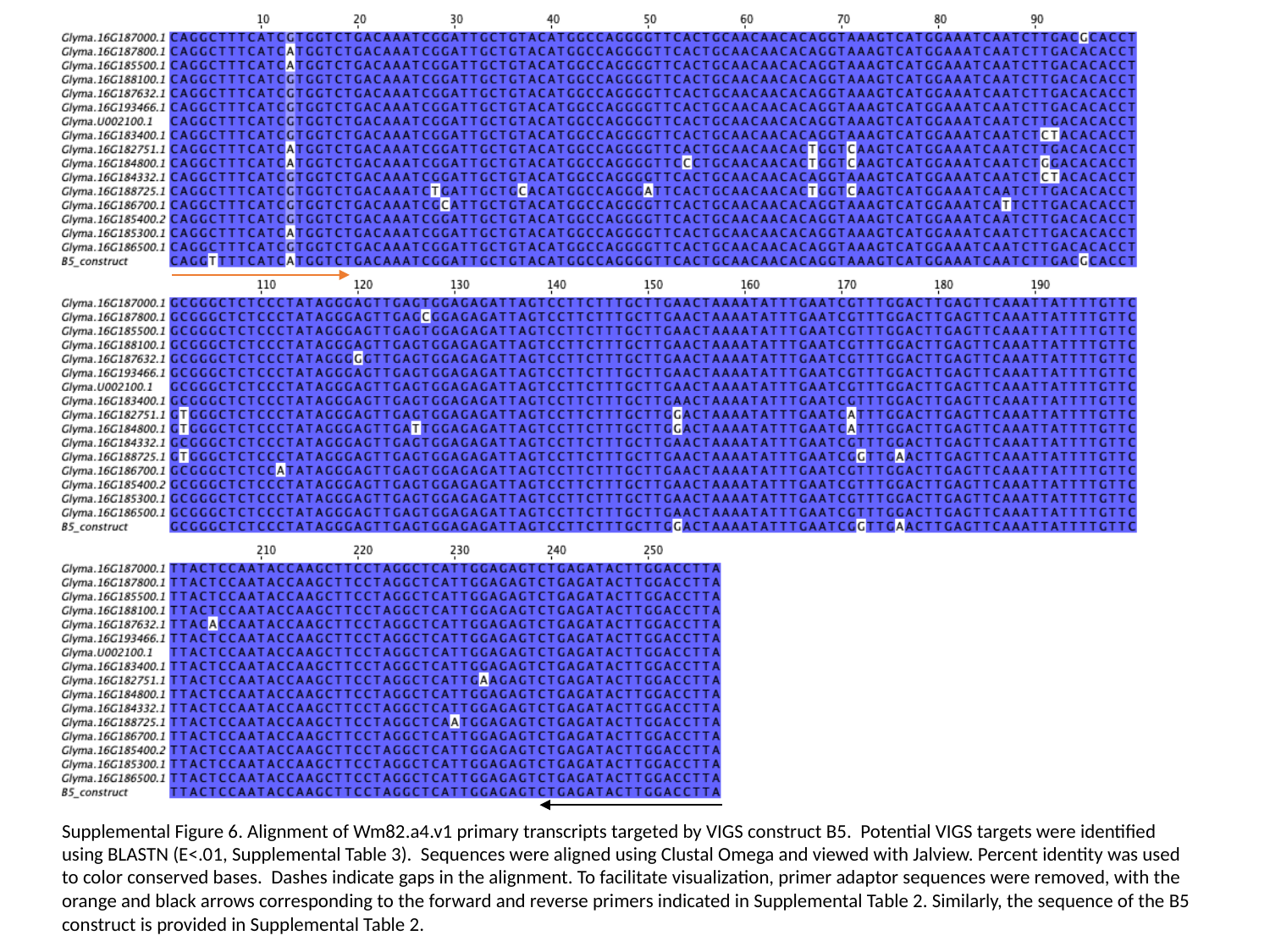

Supplemental Figure 6. Alignment of Wm82.a4.v1 primary transcripts targeted by VIGS construct B5. Potential VIGS targets were identified using BLASTN (E<.01, Supplemental Table 3). Sequences were aligned using Clustal Omega and viewed with Jalview. Percent identity was used to color conserved bases. Dashes indicate gaps in the alignment. To facilitate visualization, primer adaptor sequences were removed, with the orange and black arrows corresponding to the forward and reverse primers indicated in Supplemental Table 2. Similarly, the sequence of the B5 construct is provided in Supplemental Table 2.

## Slide 7
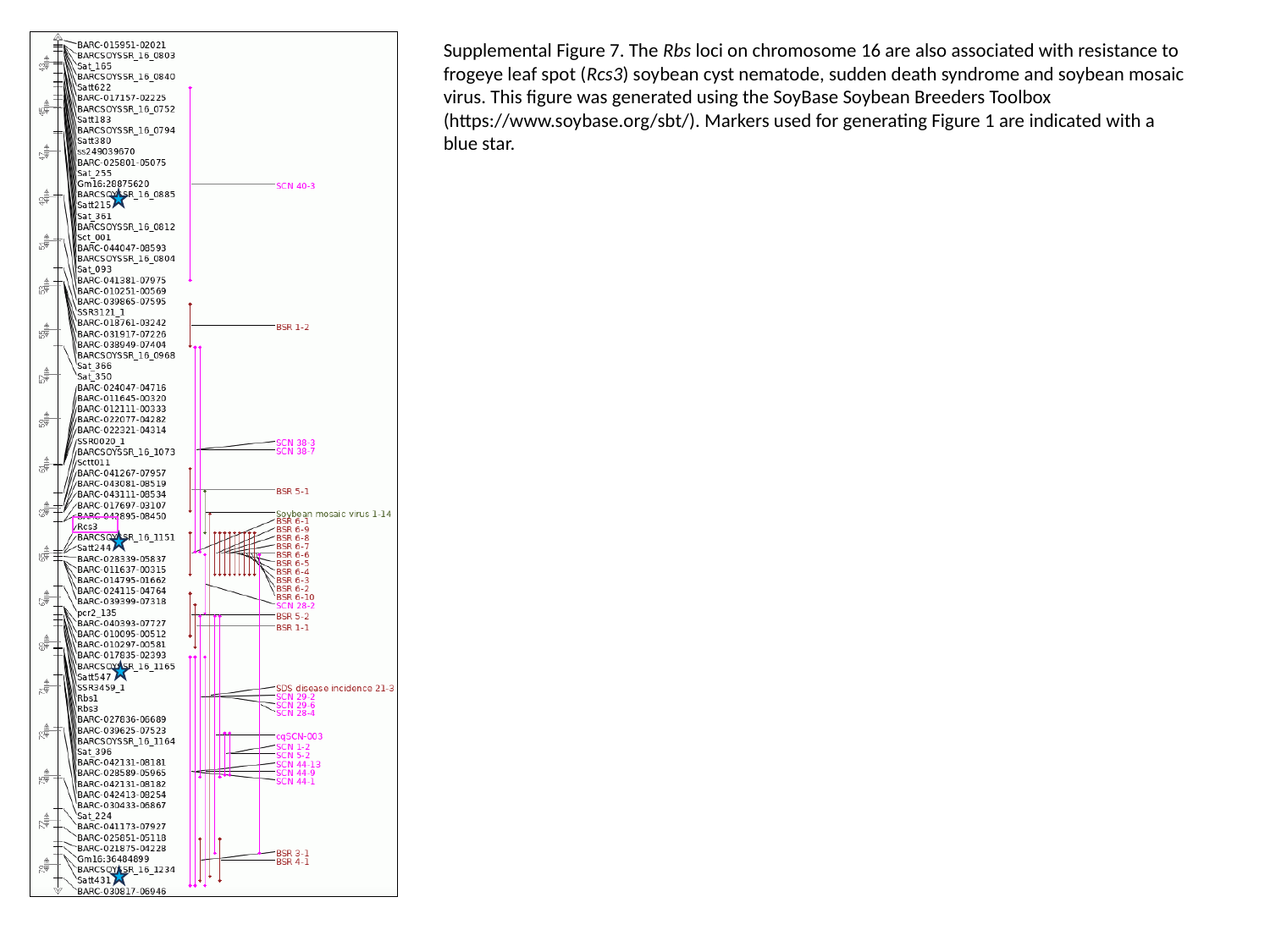

Supplemental Figure 7. The Rbs loci on chromosome 16 are also associated with resistance to frogeye leaf spot (Rcs3) soybean cyst nematode, sudden death syndrome and soybean mosaic virus. This figure was generated using the SoyBase Soybean Breeders Toolbox (https://www.soybase.org/sbt/). Markers used for generating Figure 1 are indicated with a blue star.
